# A Novel Matrix Protein, PfY2, Functions as a Crucial Macromolecule during Shell Formation

**DOI:** 10.1038/s41598-017-06375-w

**Published:** 2017-07-20

**Authors:** Yi Yan, Dong Yang, Xue Yang, Chuang Liu, Jun Xie, Guilan Zheng, Liping Xie, Rongqing Zhang

**Affiliations:** 1 Institute of Marine Biotechnology, School of Life Sciences, Tsinghua University, Beijing, 100084 China; 2 Protein Science Laboratory of the Ministry of Education, Tsinghua University, Beijing, 100084 China; 3 Department of Biotechnology and Biomedicine, Yangtze Delta Region Institute of Tsinghua University, Jiaxing, 314000 China

## Abstract

Biomineralization, including shell formation, is dedicatedly regulated by matrix proteins. PfY2, a matrix protein detected in the ethylene diamine tetraacetic acid (EDTA)-soluble fraction from both prismatic layer and nacreous layer, was discovered by our group using microarray. It may play dual roles during biomineralization. However, the molecular mechanism is still unclear. In this research, we studied the function of PfY2 on crystallization *in vivo* and *in vitro*, revealing that it might be a negative regulator during shell formation. Notching experiment indicated that PfY2 was involved in shell repairing and regenerating process. Repression of PfY2 gene affected the structure of prismatic and nacreous layer simultaneously, confirming its dual roles in shell formation. Recombinant protein rPfY2 significantly suppressed CaCO_3_ precipitation rate, participated in the crystal nucleation process, changed the morphology of crystals and inhibited the transformation of amorphous calcium carbonate (ACC) to stable calcite or aragonite *in vitro*. Our results may provide new evidence on the biomineralization inhibition process.

## Introduction

Biomineralization is an extremely widespread phenomenon, and over 60 different biominerals have been identified in organisms^1, 2^. They play multiple roles in feeding, defensing, supporting and moving^3–9^. Mollusk shell and pearl are typical biominerals which have always been the subjects of much interest in material and biological science because of their vital function for bivalve’s survival and unusual mechanical properties. Calcium carbonate (CaCO_3_), the major components of shell and pearl, mainly exists as calcite, aragonite, vaterite or amorphous calcium carbonate (ACC) which is usually considered as the precursor in biomineralization^3–5^. Organisms control the biomineralization process by the regulation of the formation, stabilization, destabilization and transformation of ACC^3, 6, 10^.

Pearl oysters, *Pinctada fucata*, famous as important economical pearl production species, are one of the best studied biomineralization models^8^. Their shells are composed of three distinct layers: the surface layer, periostracum and two other calcified layers, the prismatic and nacreous layers, where CaCO_3_ crystals are deposited as calcite and aragonite respectively^7, 9^. The main component of the shells is CaCO_3_, and the organic macromolecules, including matrix proteins, polysaccharides and lipids, only account for less than 5% (w/w). These macromolecules, particularly the matrix proteins, are quite crucial for the regulation of shell formation by playing important roles in crystal nucleation, crystal orientation, crystal polymorphism and crystal morphology^11–13^. For example, protein PfN23 accelerates the deposition of CaCO_3_ and induces the formation of aragonite crystals^14^. Aspein controls the CaCO_3_ crystal polymorph (calcite and aragonite) *in vitro* ^15^. ACC Binding Protein (ACCBP) involved in ACC formation and stabilization induces aragonite formation via an ACC precursor in low Mg/Ca ratio solutions at low temperature^16^.

Nearly 50 different kinds of shell matrix proteins have been identified and reported until now^11^. Shell matrix proteins share some significant characteristics. Firstly, they are secreted by the mantle tissue covering the inner surface of the shell, which means they have signal peptides^8^. What’s more, their primary structures usually contain some modular structures or tandem-arranged repeat units^17^. There are several traditional ways to obtain matrix proteins. Biochemistry extracting methods were initially applied to isolate them, however, only those with high abundancy could be discovered^18–22^. The polymerase chain reaction (PCR) and rapid amplification of cDNA ends (RACE) techniques were used to screen new matrix protein members based on available protein sequences. Lysine (K)-rich matrix protein (KRMP) family and Prisilkin-39 were confirmed via these molecular biology and biochemistry measures mentioned above^23, 24^. They are typical cases with the deficiency that only proteins similar to previously identified ones could be found.

New methods have been applied to explore matrix proteins based on ontogenetic development. The life cycle of *P*. *fucata* is divided into six stages, including the fertilized egg, trochophore stage, D-shaped stage, umbonal stage, juvenile stage and adult^25^. Prodissoconch I, forming during the early D-shaped stage, is the original form of the shell, consisting essentially of ACC crystalline form, which is also known as the precursor of aragonite and calcite^26, 27^. Prodissoconch II with a homogeneous structure of aragonite and a thin structure of calcite appears at the lateral D-shaped stage or umbonal stage. It is not until the juvenile stage has *P*. *fucata* formed the comprehensive prismatic and nacreous layers^26^. Our global gene expression analysis profiles during larval development showed that most genes involved in biomineralization, including nacrein, pearlin, Pif, ACCBP, Prisilkin-39, Shematrin and KRMP family, were greatly up-regulated in juveniles and maintained a relative high expression level^17^. Besides, some secreted peptides with tandem-arranged repeat units sharing similar expression patterns implied the existence of a group of undiscovered matrix protein candidates still in the need of investigations.

In the present work, we named and investigated one matrix protein PfY2, revealing its noticeable effects on CaCO_3_ crystallization and shell formation. Quantitative PCR and *in situ* hybridization analysis suggested PfY2 might influence the formation of both prismatic layer and nacreous layer. Purified recombinant rPfY2 was characterized and assessed by biochemical analyses to investigate its function *in vivo* and *in vitro*. Those results may provide us deeper insights into the control of shell formation process and the perspective of the synthesis of artificial nacre.

## Methods

### Ethics statement

This study was approved by the Animal Ethics Committee of Tsinghua University, Beijing, China.

### Animals

The adult pearl oysters, live and healthy *P*. *fucata*, with a shell length 5–6 cm and 30–40 g of wet weight, were purchased from the Zhanjiang Pearl Farm (Guangdong, China). They were maintained in glass aquaria filled with aerated artificial seawater (Sude Instant Sea Salt, 3%) for a week under controlled temperature (20 ± 2 °C) before experiments. They were fed with spirulina powder every day.

Polyclonal antibodies against PfY2 were raised in New Zealand rabbits following standard immunization procedures. All rabbits were raised under standardized pathogen-free conditions in the Animal Care Facility at Beijing. The study protocol for the experimental use of the animals was approved by the Ethics Committee of National Center for Clinical Laboratories. The 3R principles of animal experimentation (reduction, replacement, and refinement) were strictly followed.

### Isolation and identification of gene PfY2

#### RNA extraction from *P*. *fucata*

Total RNA of the tissues from different individuals (5 individuals in every single group in RNAi experiments and 4 individuals in shell notching experiments in expression analyses of PfY2) were extracted using the TRIzol reagent (Invitrogen, USA). RNA integrity was determined by fractionation on a 1.2% formaldehyde denatured agarose gel stained with Goldview (Solarbio, China). The quality and quantity of RNA was determined by measuring the OD_260/280_, OD_260/230_ and OD_260_ with a NanoDrop Lite spectrophotometer (Thermo Scientific, USA).

#### Primers

Refer to Table 1 for primer details.

**Table 1:** Primers used in this study.

Primers used for RACE	
Long UPM	CTAATACGACTCACTATAGGGCAAGCAGTGGTATCAACGCAGAGT
Short UPM	CTAATACGACTCACTATAGGGC
NUP	AAGCAGTGGTATCAACGCAGAGT
Y2-R1	ACTTTTACTTTCAGAAGCGCAAGTCAATTC
Y2-R2	TGAGTAACACGGCAAATAGGACTGCTAC
Y2-F1	ATGAAGTCAGCTACGGTAGCAGTC
Y2-F2	CGCTTCTGAAAGTAAAAGTGTTCATCGCC
Y2-confirm F	TACGATTCCCTGTGAAAGACAATCAAACG
Y2-confirm R	TATTCTCATCACAACAAAGGCACACAGGC
**Primers cloning PfY2 to the expression vector pET**-**28a for polyclonal antibody preparation**	
eY2-F1	GGAATTCCATATGAATCCTTGGAATTATCCAGG
eY2-R1	CCGCTCGAGCTAGTTGTTCCAGTTATTCC
**Primers cloning PfY2 to the expression vector pMAL**-**c5x**	
eY2-F2	GGAATTCCATATGAATCCTTGGAATTATCCAGG
eY2-R2	GGAATTCCTAGTGATGATGATGATGATGGTTGTTCCAGTTATTCC
**Primers used for Real**-**time PCR analysis**	
RT-Y2-F1	ATGAACATGGGTAGAGAGTTC
RT-Y2-R1	CACCAGTTGTTCCTGTTCCA
RT-GAPDH-F	GCCGAGTATGTGGTAGAATC
RT-GAPDH-R	CACTGTTTTCTGGGTAGCTG
RT-KRMPI-F	AGAATGAAGTTCGCCGC
RT-KRMPI-R	GCATTTCCAATCCCAGG
RT-Nacrein-F	GGCGACGAACCGGATGACG
RT-Nacrein-R	GACTCTGTACACGGGGGAGTG
RT-P14-F	TAAGACCATTCTACGGC
RT-P14-R	ACCATATCCTCCATATAATCCT
RT-Aspein-F	TGATAGTGAAGACGATGA
RT-Aspein-R	TGTCATCATCATCATCATC
**Primers used for RNAi assay**	
dsY2-F	GCGTAATACGACTCACTATAGGGAGATTGCCGTGTTACTCACAGCGT
dsY2-R	GCGTAATACGACTCACTATAGGGAGACTACCCATGTTCATGTTCCTGC
dsGFP-F	GCGTAATACGACTCACTATAGGGAGAATGGTGAGCAAGGGCGAGGAG
dsGFP-R	GCGTAATACGACTCACTATAGGGAGATTACTTGTACAGCTCGTCCATG
**Primers used in** ***in situ*** **hybridization**	
ISTY2-F	ATGAACATGGGTAGAGAGTTCTT
ISTY2-R	CACCAGTTGTTCCTGTTCCAAGG

Abbreviation: F, forward; R, reverse; RT, Real-time qPCR; ds, double strand; IST, *in situ* hybridization.

#### Cloning and bioinformatics analyses of the complete PfY2 cDNA sequence

The extracted mantle RNA (1 μg) was used to synthesize single-stranded cDNA for RACE following SMARTer RACE cDNA Amplification Kit (Clontech, Japan). Primer Y2-R1, Y2-R2 were used with primers supplied with the 5′RACE kit. Primer Y2-F1, Y2-F2 were used with primers supplied with the 3′RACE kit. The full-length cDNA sequence was confirmed using primers Y2-comfirm F and Y2-confirm R. The deduced amino acid sequence of the gene was determined bioinformatically via the ExPASy Translate tool. The signal peptide was predicted by the SignalP 3.0.

### Expression and distribution pattern of PfY2

#### PfY2 expression analyses by Real-time PCR

500 ng total RNA in every following tissue, including foot, gonad, gill, mantle pallial, mantle edge, viscus and adductor muscle, from four individuals was reverse-transcribed into cDNA templates for RT-PCR using PrimeScript™ RT Master Mix (Perfect Real Time) (Takara, Japan) to detect the expression and distribution pattern of PfY2 in *P*. *fucata* by SYBR Premix Ex Taq (Takara, Japan), following the manufacturer’s instructions in a LightCycler^®^ 480 system (Roche Diagnostics, Switzerland). RT-PCR was conducted using the primer pairs RT-Y2-F1/RT-Y2-R1 and RT-GAPDH-F/RT-GAPDH-R to amplify PfY2 and GAPDH (included as a positive control for cDNA preparations) gene fragments, respectively. GAPDH was the internal control. To avoid false-positive results and detect cross-contamination of the samples, a negative control was performed in the absence of the cDNA template. All PCR products were subcloned and verified by sequencing. Cycle threshold (Ct) values were calculated in each reaction and normalized to an internal control (GAPDH), and relative gene expression was calculated using the comparative Ct method^28, 29^.

#### *In situ* hybridization

*In situ* hybridization was performed as described below with some modifications^30^. The mantle tissue was removed and immediately fixed in 4% paraformaldehyde containing 0.1% DEPC (Sigma-Aldrich, USA) for 1.5 days. Leica freezing microtome (Leica Biosystems CM1950) was used to cut mantle frozen sections. The fragments of PfY2 was amplified using the primers ISTY2-F and ISTY2-R in Table 1 and then subcloned into pMD-19T vector (Takara, Japan). Digoxigenin-labeled RNA probes were synthesized by DIG RNA Labeling kit (Roche Diagnostics, Switzerland). The Enhanced Sensible ISH Detection Kit II (AP) (Boster, China) was used to perform the *in situ* hybridization at room temperature.

### Identification of PfY2 as a matrix protein

#### Vector construction

The open reading frame (ORF) sequence of target gene *PfY2* without signal peptide was introduced into a pET-28a (+) vector (Invitrogen, USA) under the control of a strong T7 promoter, resulting in pET28-rPfY2 with a His_6_-tag only at the N-terminus (NH-rPfY2). The DNA sequence of recombinant protein was confirmed by direct sequencing, identical to that of authentic PfY2. The purified recombinant PfY2 with a His_6_-tag in this plasmid was used for the synthesis of polyclonal antibody.

Primers eY2-F2/eY2-R2 were used to amplify the coding region of *PfY2* without signal peptide. We added a His_6_-tag in primer eY2-R2. Then this PfY2 with a C-His tag was inserted downstream from the malE gene of *E*. *coli*., which encodes maltose-binding protein (MBP), in the pMAL-c5X vector (New England BioLabs Inc., NEB; USA), facilitating the expression of “soluble” fusion protein MBP-tagged PfY2, rPfY2. rPfY2 was purified for further functional analyses.

#### Identification of PfY2 as a shell matrix protein

EDTA-soluble and EDTA-insoluble matrices from different shell layers were prepared as described by Kong *et al*. with some modifications^24^. After removing the impurities by centrifuging for 45 min at 13,000 rpm at 4 °C the supernatant described above were concentrated by 3 kDa centrifugal ultrafiltration tubes (Amicon Ultra-4, UFC800324). The amount of the protein was determined by the Pierce bicinchoninic acid (BCA) Protein Assay kit (Thermo Scientific, USA) according to the manufacturer’s instructions. The identification of PfY2 in different extracts (EDTA-insoluble matrices and EDTA-soluble matrices from prismatic layer, EDTA-insoluble matrices and EDTA-insoluble matrices in nacreous layer) of the shell was confirmed by Western Blotting.

### Recombinant protein expression and purification

*E*. *coli* (Transsetta, DE3) (Transgene, China) was transformed with recombinant plasmid pET28-rPfY2 for the over expression of NH-rPfY2. The recombinant cells were induced by 0.45 mM isopropyl 1-thio-β-D-galactopyranoside (IPTG; Sigma-Aldrich) at 37 °C for 24 h when the cell density (OD_600_) reached 0.9. After 24 h of cultivation, cells were harvested and resuspended with lysis buffer (20 mM Tris, 500 mM NaCl, 10% glycerol and 20 mM imidazole; pH 7.5). Then cells were disrupted via ultrasonic dismembrator (Sonics & Materials Inc., USA) at 28% power with 4 s pulse on and 6 s pulse off repetition cycle on ice. After centrifugation 12000 g for 40 min at 4 °C, the supernatant was removed and the insoluble fraction was used for purification and antibody preparation. The insoluble precipitation was re-dissolved and resuspended in denaturing lysis buffer (20 mM Tris, 500 mM NaCl, 6 M urea; pH 7.5). After the same centrifugation condition, the urea-soluble supernatant was collected and mixed with Ni-nitrilotriacetic acid (Ni-NTA) resin (CWBIO, China). The mixture was incubated at 4 °C for 1 h and then washed by washing buffer (20 mM Tris, 500 mM NaCl, 6 M urea, 40 mM imidazole; pH 7.5) to remove the heteropolymeric protein. NH-rPfY2 was then eluted in elution buffer (20 mM Tris, 500 mM NaCl, 6 M urea, 200 mM imidazole; pH 7.5). Then the protein gel was injected into New Zealand rabbits. Anti-PfY2 polyclonal antibodies were raised in these rabbits following standard immunization procedures according to the manufacturer’s instructions.

The purification measures and methods of MBP-tagged PfY2 (The recombinant protein rPfY2-MBP with a His_6_ tag on C-terminal, rPfY2) were similar. The IPTG inducing condition was changed for 15 °C 12 h. All the buffer used excluded urea while the remaining components were the same. The soluble, active rPfY2 was eluted using elution buffer (20 mM Tris, 500 mM NaCl, 300 mM imidazole; pH 7.5) then desalted using a Hitrap desalting column (GE Healthcare, USA) to storage buffer (20 mM Tris, 500 mM NaCl; pH 7.5) and stored at 4 °C for further analyses and experiments. The purification of MBP was performed according to the manufacturer’s instructions (GE Healthcare, USA).

### Functions of PfY2 in shell reparation and formation

#### Shell notching

Shell notching assays were performed following the literature with some modifications^31^. Pearl oysters were randomly divided into 8 groups with 5 individuals each. The age, weight and body size of every individual in every group were almost the same. A V-shape notch was cut on the shell margin near the adductor muscle without touching the mantle tissue. Mantle tissues were removed into liquid nitrogen at 0, 6, 12, 24, 36, 48, 72, 96 h after notching and total RNA from all groups were extracted at 96 h. Mantle tissues from five oysters without any treatment were used as controls. RT-PCR was then performed to detect the expression level of related genes. Matrix protein KRMP and nacrein were positive controls. Nacrein contains two carbonic anhydrase domains, responsible for catalyzing the formation of hydrogen carbonate from water and carbon dioxide^20^. That’s the reason why nacrein must accumulate in the shell to provide plenty of hydrogen carbonate for crystal and shell formation although nacrein actually depressed CaCO_3_ crystallization^20, 32^. EDTA-insoluble matrix protein KRMP3 participated in the framework formation of the shell^23^. Therefore, nacrein and KRMP3 were chosen as positive controls in this notching assay. All experiments were repeated three times.

#### Silencing of matrix protein PfY2

RNAi assays were performed as described by Suzuki *et al*. with some modifications^33^. Specific primers dsY2-F and dsY2-R were designed according to PfY2 coding region. Vector pEGF-N1 (NEB, USA) was used as template to amplify GFP. PCR products were purified by EasyPure Gel Purification Kit (Transgene, China). Double strand RNAs were synthesized using RiboMAX™ Large Scale RNA Production System T7 kit (Promega, USA). They were diluted to 60 μg/100 μl or 120 μg/100 μl in DNase and RNase free water (TIANGEN, China) and then injected into the adductor muscle of *P*. *fucata*. Every treatment group contained five 2-year-old oysters. Equivalent amount of GFP dsRNA and DNase and RNase free water were injected in control group, respectively. All oysters in every group were sacrificed six days after injection and total RNA were extracted from the mantle tissues of each oyster to detect the RNAi efficiency by RT-PCR (see Table 1 for primers). Dissociation curves were investigated to determine product purity and amplification specificity. Samples were normalized to 100 μl of sample from the H_2_O-injected group, which was assigned a relative value of 1.0. RNAi and other experiments were repeated twice. All of the shells were washed and saturated in Milli-Q water, then air dried and cut into small sections. After that, all of them were examined and observed through scanning electron microscope (SEM; FEI Quanta, 15 kV) after coating with gold. At least 20 images were taken from each shell, and typical images were chosen to present the results.

### Functions of PfY2 on calcium carbonate crystallization

#### Calcium carbonate binding assay

Calcite (Alfa Aesar, USA) and aragonite (Alfa Aesar, USA) powder were used as supplied. Specifically, 0.2 g calcite/aragonite powder was suspended in 600 μl protein storage buffer (20 mM Tris, 500 mM NaCl; pH 7.5) by vigorous stirring then the mixture was gently rocked at 4 °C overnight. The concentration of every kind of protein, BSA, rMBP and rPfY2, was 40 μg/ml, respectively. After 12 h incubation, the calcite/aragonite would be washed by Milli-Q deionized water and protein storage buffer three times each and the fluids flowing through was collected to detect the binding properties of different proteins. Then the powder-protein mixture in every group was collected separately and 120 μl loading buffer (40 mM Tris, 2% SDS, 10% glycerol, 1% β-mercaptoethanol, and 0.004% bromophenol blue; pH 6.7) was added to each group. After being boiled at 100 °C for 10 min, SDS-PAGE was performed to analyze the binding abilities of rPfY2, MBP and BSA.

#### *In vitro* calcium carbonate crystallization assay

The preparation of saturated calcium bicarbonate solution was executed in terms of ref. 34 with some modifications. CaCO_3_ (Sigma-Aldrich, 0.15 g) was dissolved in 20 ml of Milli-Q water and CO_2_ gas was bubbled into this solution for five hours. Then the solution was filtered by a 0.22 μm MILLEXGP filter unit (SLGP033RB) to remove the excess solid CaCO_3_. The filtrate was aerated with CO_2_ for another two hours before immediate use. The only difference between calcite and aragonite crystal system *in vitro* was the induction of magnesium chloride. The concentration of Mg^2+^ in aragonite system was 50 mM. Crystallization experiments were carried out by adding bovine serum albumin (BSA; Sigma-Aldrich), recombinant rPfY2 and MBP to a final volume of 20 μl Ca(HCO_3_)_2_ respectively and incubated for 48 h on a siliconized cover glass placed in a wet box. The volume of the proteins added didn’t exceed 2.5 μl in every single group. The final concentration of rPfY2 (the recombinant protein PfY2-MBP with a His_6_ tag on C-terminal) was 30 μg/ml, 50 μg/ml and 80 μg/ml in three independent experimental groups separately. The final concentration of BSA and MBP was 80 μg/ml each. After 48-hour incubation, all the cover glasses were gently washed by Milli-Q deionized water three times and the crystals were naturally dried before observation. All the experiments were repeated three times.

#### Calcium carbonate precipitation rate inhibition assay

The inhibitory effects of rPfY2 on calcium carbonate precipitation rate was assessed by a spectrophotometer (Bio-rad 680, USA) in reference to Suzuki’s methods with minor modifications^35^. Different concentrations of CaCl_2_ and NaHCO_3_ were prepared and filtered by a 0.22 μm MILLEXGP filter unit. rPfY2 was mixed with CaCl_2_ (Sigma-Aldrich; 100 μl, 100 mM) in a 96-well COSMO plate. Then the NaHCO_3_ (Sigma-Aldrich; 100 μl, 100 mM) was quickly added to above mixtures. The final concentration of rPfY2 was 2 μg/ml, 5 μg/ml and 10 μg/ml in experimental groups. rMBP and BSA were 10 μg/ml each. The precipitation rate of CaCO_3_ was measured every minute for 6 min using a spectrophotometer (Bio-rad 680). All experiments were taken three times independently and repeated three times. The crystals were observed and characterized after 24 h.

#### Transition of amorphous calcium carbonate to stable crystals

100 μg/ml rPfY2 was added into 50 mM CaCl_2_, prepared as solution A. 50 mM Na_2_CO_3_ was prepared as solution B. Solution A and B were cooled on ice for 1 h and then an equal volume of solution B was quickly added to solution A and mixed well at 4 °C. After 4 h and 24 h, crystals were collected and washed by ethanol for three times, dried and analyzed by X-ray diffraction (XRD). In the system generated by the induction of ACC to aragonite, 100 μg/ml rPfY2 with 100 mM MgCl_2_ was added to 50 mM CaCl_2_ as solution A^10, 16^. The remaining steps were exactly same as mentioned above. rMBP and BSA were introduced into the same transition system as controls.

### Crystal observation and characterization

The morphologies of the crystals were examined by SEM (FEI Quanta). The crystal form was characterized by Raman spectrum and XRD. Raman spectroscopy was performed with an excitation wavelength of 514 nm provided by a Renishaw RM2000 spectrometer and the crystals were scanned for 90 s from 100 to 1900 cm^−1^ 3 times for specific identification. XRD analyses were performed on a D8 ADVANCE (Bruker, Germany) X-ray diffractometer over the 2θ range 10–90°.

### Statistical analyses

All figures were created using SigmaPlot 11.0 (Systat Software Inc., Germany) and Photoshop CC 2015 (Adobe, USA). The significance differences were calculated by Student’s t test and Ducan’s new multiple range method using the mixed procedure of the SAS version 8.0 software package (SAS Institue Inc., USA).

## Results

### Identification and bioinformatics analyses of PfY2

**Figure 1:**
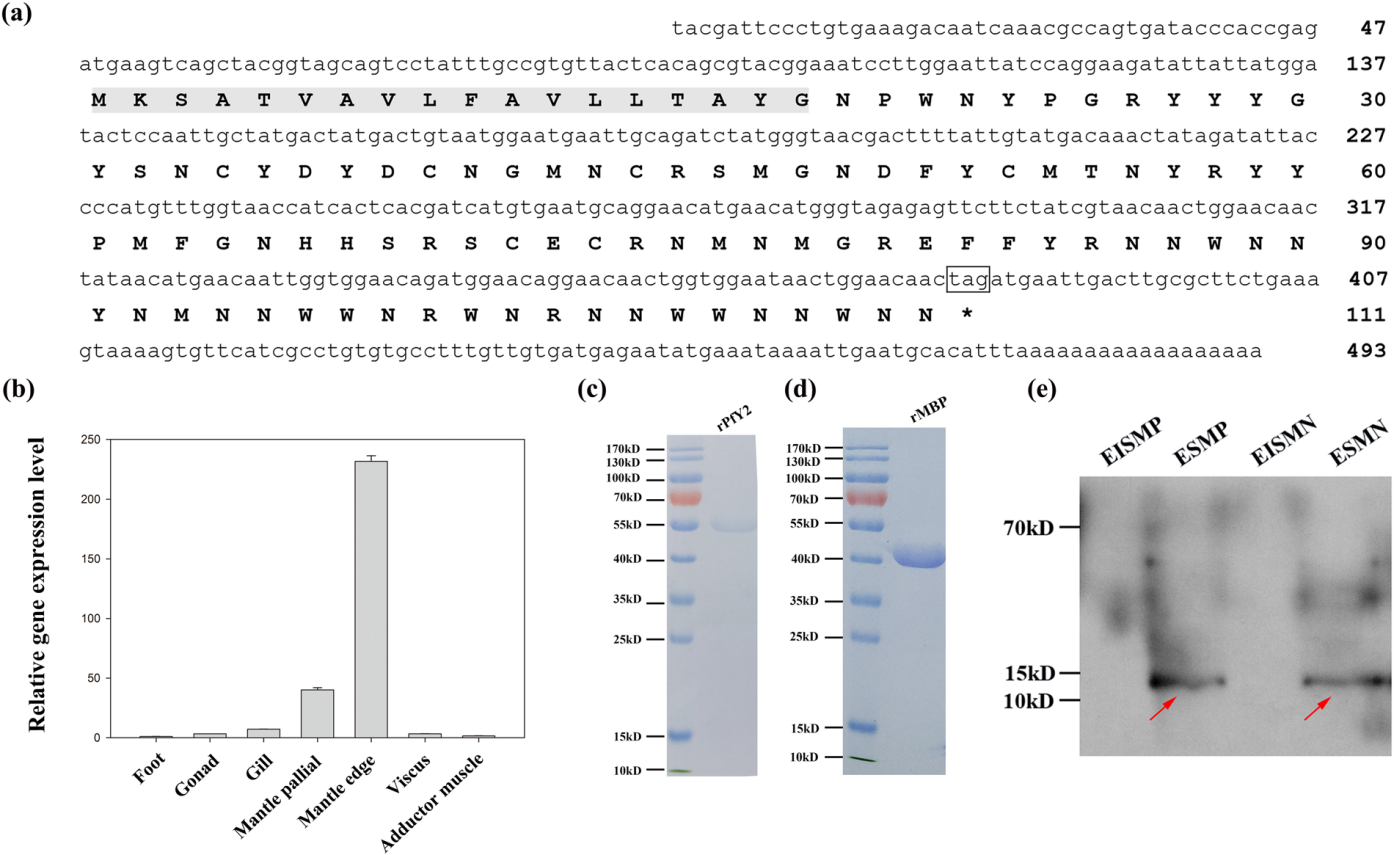
Identification of PfY2 as a matrix protein. (**a**) The cDNA sequence information of PfY2. The shaded section is the signal peptide. Black box is the transcription termination site. (**b**) Expressional analyses of PfY2 in various tissues by RT-PCR. Values for RT-PCR are means ± standard deviation of three replicates. (**c**) SDS-PAGE of recombinant protein PfY2 with MBP tag at 55 kD. (**d**) SDS-PAGE of recombinant protein MBP tag at 40 kD. (**e**) Detection of native PfY2 in the shell via Western Blotting. The red arrow indicated the band of the native PfY2 in shell components. EISMP, EDTA-insoluble fraction from prismatic layer; ESMP, EDTA-soluble fraction from prismatic layer; EISMN, EDTA-insoluble fraction from nacreous layer; ESMN, EDTA-soluble fraction from nacreous layer. Please refer to Suppl. Fig. S4 to get the primary gel pictures.

Current findings suggest that most known matrix proteins and proteins related to shell formation could be up-regulated by at least 20-fold from the umbonal stage to juveniles^17^. The full length of PfY2 (GenBank^TM^ accession number KY436033) cDNA sequence is 493 bp, including a 5′-untranslated region of 47 bp, an ORF of 336 bp encoding a deduced 111-amino acid protein, and a 3′-untranslated region of 110 bp (Fig. 1a). PfY2 was increased by 152-fold during the transition from umbonal stage to juveniles (Suppl. Fig. S1).The BLASTx search against the GenBank^TM^ nr database revealed that PfY2 showed no similarities to any genes in any known species, and the function of PfY2 still remained unknown. The deduced mature protein had a calculated molecular mass of 11.9 kDa.

### The expression and distribution pattern of PfY2

On one hand, for better understanding and evaluating the potential functions of PfY2 protein, the tissue-specific expression of PfY2 was detected by RT-PCR in eight tissues, including foot, gonad, gill, mantle pallial, mantle edge, viscus and adductor muscle. RT-PCR results showed that PfY2 exhibited the highest expression level in mantle edge, followed by mantle pallial (Fig. 1b). It is generally agreed that mantle tissue is responsible for shell formation. And the mantle edge is closely related with prismatic layer, whereas the mantle pallial is reported to be involved in the formation of nacreous layer^36, 37^.

On the other hand, *in situ* hybridization was performed as described above to localize the specific expression site of PfY2 mRNA in mantle tissue of frozen sections. The hybridization signals were detected in the epithelial cells of both the outer fold and middle fold (Suppl. Fig. S2b). The control group showed no significant signals (Suppl. Fig. S2a). This result was also consistent with the RT-PCR detection analyses.

In general, PfY2 specifically expressed in the mantle tissue of *P*. *fucata* and was very likely involved in the formation of both nacreous layer and prismatic layer.

### Expression and purification of recombinant protein rPfY2 and maltose binding protein rMBP

Considering the relative low expression efficiency of native PfY2, its recombinant expression *in vitro* seems to be a viable alternative to characterize. The recombinant protein was sufficiently pure for *in vitro* function studies (Fig. 1c,d). The molecular mass of rPfY2 (55 kD) is almost identical to calculated value (PfY2 with His-tag 12 kD plus MBP 40 kD), indicating that rPfY2 was not highly glycosylated or phosphorylated. What’s more, mass spectrum was performed to confirm the protein sequence of rPfY2 (Suppl. Fig. S3). We also expressed and purified rMBP as a negative control (Fig. 1d) for further function analyses. In this research, the recombinant protein MBP-PfY2 with a His_6_-tag on C-terminal, rPfY2, was used for further function analyses.

### Identification of PfY2 as a matrix protein in *P*. *fucata* shell

Gene expression pattern analyses were not enough to confirm that native PfY2 protein truly existed in either the nacreous layer and/or the prismatic layer. As a result, we used the polyclonal antibody against recombinant protein PfY2 for immunodetection of native PfY2. As we can see from Fig. 1e, PfY2 could be detected in EDTA-soluble matrixes from both nacreous and prismatic layers by Western Blotting. In controls, no signal was detected in the absence of the anti-PfY2 (Suppl. Fig. S5).

### *In vivo* function analyses of PfY2 during shell reparation and formation

**Figure 2:**
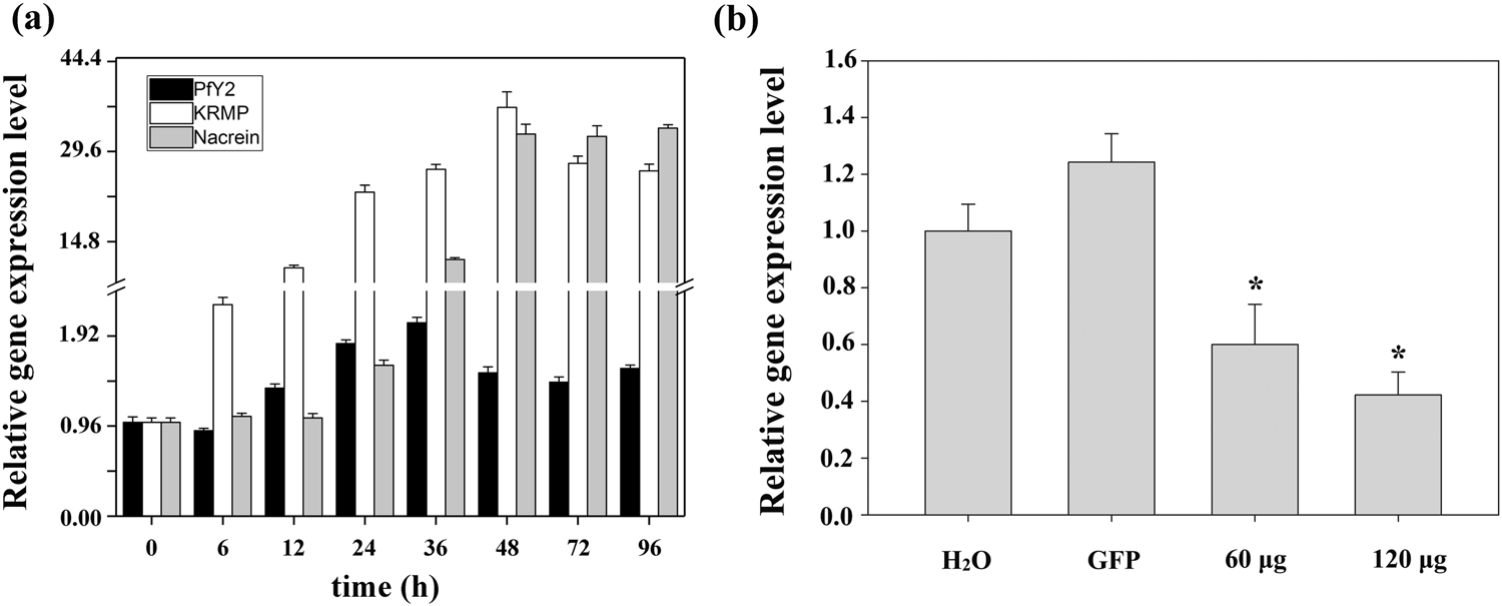
*In vivo* functions of PfY2. (**a**) PfY2 expression after shell notching. Expression of negative control (0 h) gave a relative value of 1.0. (**b**) PfY2 expression inhibited by RNAi. Negative control water-injected group gave a relative value of 1.0. The oysters in non-target control group were injected with GFP dsRNA. The expression of PfY2 was significantly (*p* < 0.05) inhibited after the injection of specific PfY2 dsRNA in both 60 μg and 80 μg group compared with the oysters injected with water. Asterisk, significant difference (*p* < 0.05).

We conducted shell notching assays to investigate the roles of PfY2 during shell reparation and formation, which also contributed to better understanding the complex natural shell regeneration process. The expression level of PfY2 increased 12 h after notching, reaching the peak at 36 h. PfY2 expression increased to approximate 200% of the value before notching. Though with a slightly decreasing in the following hours, the expression of PfY2 was still up-regulated compared with the negative control. We also detected the relative gene expression level of other matrix proteins, KRMP and nacrein, after shell notching (Fig. 2a). Different from PfY2, the increasing degree of the other two protein showed was much more dramatic. KRMP was up-regulated dramatically only 6 h after notching, while nacrein’s expression had not increased until 24 h later. In addition, the expression of nacrein kept going up over time. Conversely, the expression pattern of KRMP and PfY2 was similar. They both increased shortly after notching, underwent a slight decline and finally reached a relative balance stage.

**Figure 3:**
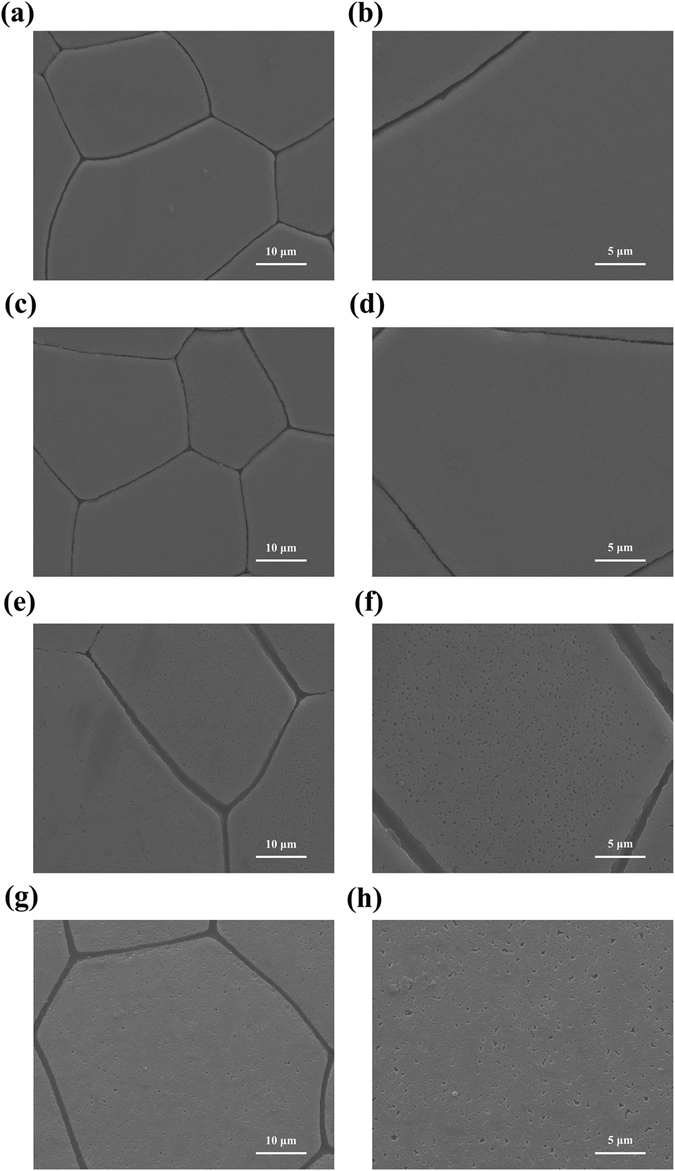
Effects of the inhibition of PfY2 on regulating the prismatic layer of inner shell. (**a**,**b**) SEM images of the normal shell prismatic layers from oysters injected with water. (**c**,**d**) SEM images of the prismatic layers in GFP-injected group shells. (**e**,**f**) SEM images of the prismatic layers in 60 μg PfY2 dsRNA injected group shells. (**g**,**h**) SEM images of the prismatic layers in 120 μg PfY2 dsRNA injected group shells. (**b**,**d**,**f**,**h**) showed an enlargement of (**a**,**c**,**e**,**g**) respectively.

**Figure 4:**
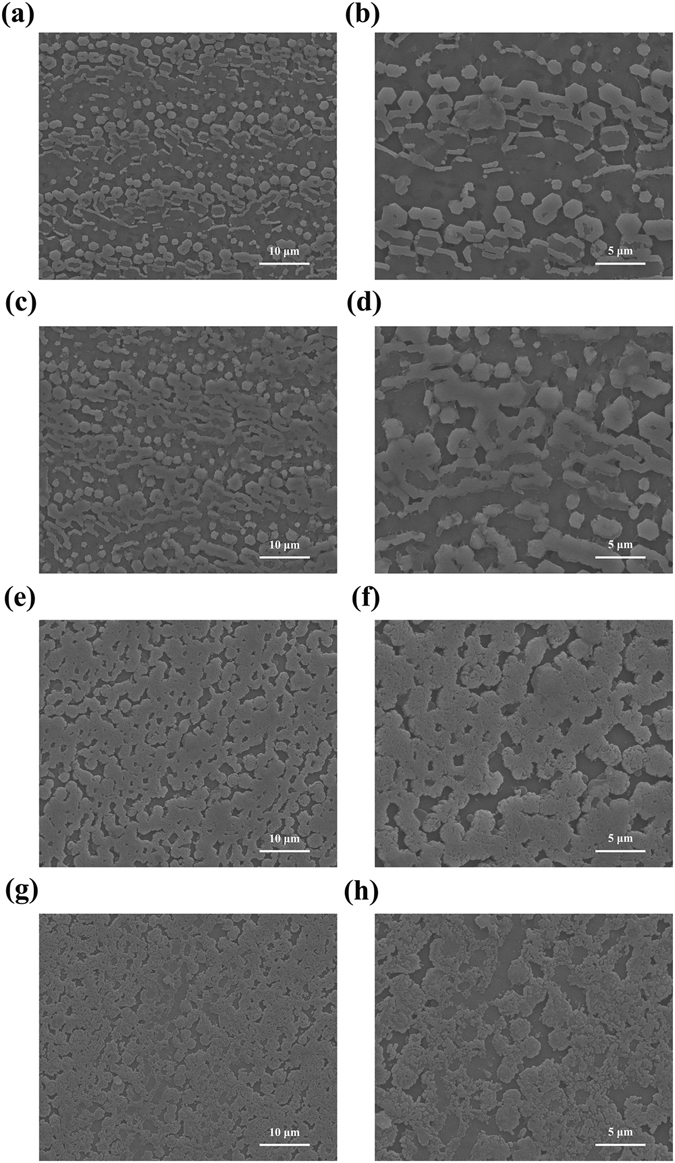
Effects of the inhibition of PfY2 on regulating the nacreous layer of inner shell. (**a**,**b**) SEM images of the normal shell nacreous layers from oysters injected with water. (**c**,**d**) SEM images of the nacreous layers in GFP-injected group shells. (**e**,**f**) SEM images of the nacreous layers in 60 μg PfY2 dsRNA injected group shells. (**g**,**h**) SEM images of the nacreous layers in 120 μg PfY2 dsRNA injected group shells. (**b**,**d**,**f**,**h**) showed an enlargement of (**a**,**c**,**e**,**g**) respectively.

The function of PfY2 during shell formation was further analyzed by RNAi. The microstructures of the shell could be disrupted after the down-regulation of certain matrix proteins. We designed the specific double strand RNAs to knock down the expression of PfY2 by injecting them into the adductor muscles of oysters. Oysters in the control groups were injected with GFP dsRNA or water only. Seven days after injection, total RNA from mantle tissues were extracted and RT-PCR was performed to detect the expression level of PfY2. The expression of PfY2 in the GFP dsRNA-injected group was similar to the water-injected group. However, the expression of PfY2 was decreased to approximate 60% in the group injected with 60 μg PfY2 dsRNA and 40% in the group injected with 120 μg PfY2 dsRNA compared with the group injected with water (Fig. 2b). The surfaces of both prismatic layer and nacreous layer in all treated groups were observed under SEM. In the control groups (GFP dsRNA-injected group and water-injected group), prismatic layer showed normal smooth surfaces (Fig. 3a–d), with the clearly visible edges and the nacreous tablets were regular hexagonal structures in nacreous layer (Fig. 4a–d). Compared with those normal phenotypes, the injection of PfY2 dsRNAs led to the disruption of microstructures in both prismatic and nacreous layer. The surface of prismatic layer became rougher and lacunose after injecting PfY2 dsRNA (Fig. 3e–h), while the morphology of the nacreous layer was irregular with the overgrowth of the crystals above the nacreous tablets supposed to grow normally (Fig. 4e–h). These changes in both prismatic layer and nacreous layer were more obvious in the 120 μg group, suggesting PfY2′s dual roles during shell reparation and formation.

### Binding properties of rPfY2 with main shell components

**Figure 5:**
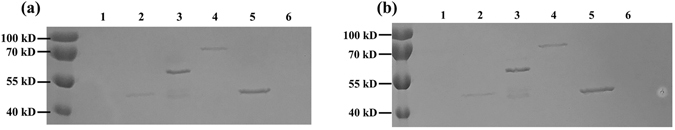
Binding properties of rPfY2 with main shell components. Lane 1–3, BSA, MBP, rPfY2 binding with calcite (**a**), aragonite (**b**) after flushing with water and protein elution buffer three times each, respectively. Lane 4–6, residual unbound BSA, MBP, rPfY2 in 1^st^ wash fraction after incubation with calcite (**a**), aragonite (**b**), respectively. Please refer to Suppl. Figs S7 and S8 to get the primary gel pictures.

Interactions with the main shell components, calcite and aragonite, are one of the most important attributes of calcification-related matrix proteins. Therefore, we investigated the binding properties of rPfY2 with calcite and aragonite for better analyzing the mechanisms of how rPfY2 affected the shell formation. rPfY2 was incubated with prepared calcite and aragonite powders overnight. The concentration of every kind of protein, BSA, rMBP and rPfY2 used in binding experiments was 40 μg/ml. No matter what crystal system rPfY2 was added, it could be detected in both calcite and aragonite crystals even all the crystals had been flushed by water and protein storage buffer three times each (Fig. 5a lane 3, Fig. 5b lane 3). Despite that rMBP (Fig. 5a lane 2, Fig. 5b lane 2) was also detected in calcites and aragonites, the amounts of which had already bound to the crystals, were much less compared with the washing fractions. Above all, the binding protein rPfY2 (Fig. 5a, lane 3) in calcite system was a lot more than the rMBP (Fig. 5a, lane 2) and the BSA (Fig. 5a, lane 1) binding to the calcium carbonate precipitations. Similar binding effects were found in aragonite crystalline system at the same time (Fig. 5b). Above all, this binding property is the foundation of matrix proteins’ regulation on crystal nucleation, crystal orientation, crystal polymorphism and morphology^11–14, 38^.

### Calcium carbonate crystallization in the presence of rPfY2 *in vitro*

**Figure 6:**
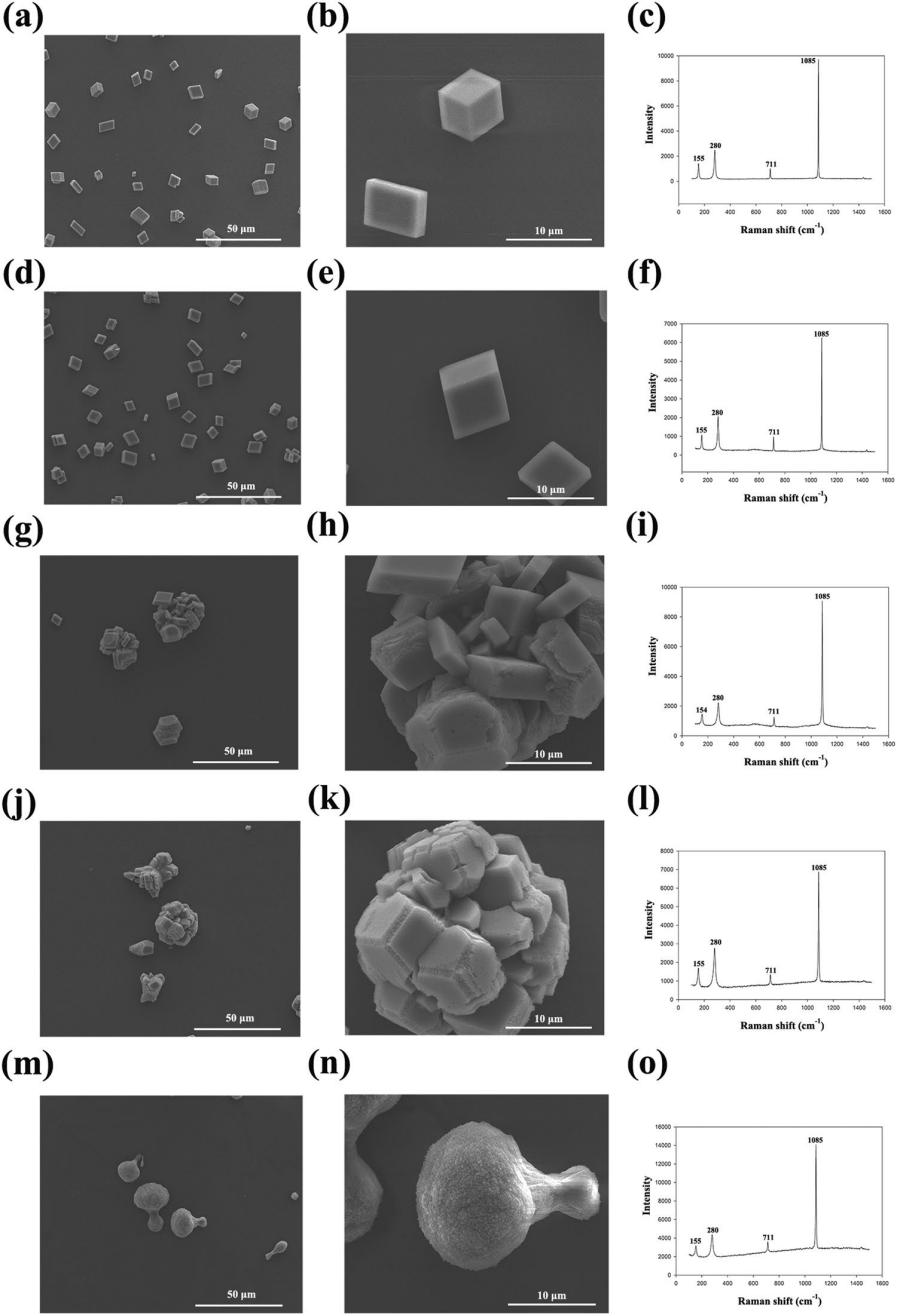
SEM images of *in vitro* calcite crystallization in the presence of rPfY2 and Raman spectrum analyses of crystals. Crystals grown in the presence of 80 μg/ml BSA (**a**,**b**); 80 μg/ml MBP (**d**,**e**); 30 μg/ml rPfY2 (**g**,**h**); 50 μg/ml rPfY2 (**j**,**k**); 80 μg/ml rPfY2 (**m**,**n**). (**b**,**e**,**h**,**k**,**n**) were the amplifications of the crystals indicated by arrows in (**a**,**d**,**g**,**j**,**m**), respectively. (**c**,**f**,**i**,**l**,**o**) showed the Raman spectrum of the crystals from 80 μg/ml BSA (**b**), 80 μg/ml MBP (**e**), 30 μg/ml rPfY2 (**h**), 50 μg/ml rPfY2 (**k**) and 80 μg/ml rPfY2 (**n**), respectively.

The shell of *P*. *fucata* is an organic mineral assemblage dominated by calcium carbonate with a minor organic matrix complex. *In vitro* calcium carbonate crystallization assay has been widely used to mimic the biomineralization process of shell formation. Firstly, we examined the influences of rPfY2 on calcite without the addition of Mg^2+^. In negative control groups, the crystals were typical rhombohedra in the presence of 80 μg/ml BSA as expected, Raman measurements indicating they were standard calcite (Fig. 6a–c). These results were consistent with our previous studies^14, 38, 39^. We also observed the morphology and detected the crystal form via SEM and Raman respectively in the MBP group to eliminate the effects of the tag (80 μg/ml) and found MBP had no effect on the crystallization of calcite either (Fig. 6e–f). In contrast, the addition of rPfY2 changed the morphologies of calcite significantly (Fig. 6g–o). With the increasing concentration of rPfY2 from 0 μg/ml to 80 μg/ml, the edges and corners of the crystals turned rounder and tended to disappear. It seemed like the calcite crystals gathered and rPfY2 might induce an integration of crystal particles to some extent. When the concentration of rPfY2 reached 80 μg/ml, the crystals were transformed into a dumbbell shape (Fig. 6m,n), which was quite similar to aragonites observed in aragonite crystallization system *in vitro*. However, the crystal form had not been changed according to Raman spectrum, with the characteristic peaks of 155, 280, 711 and 1085 cm^−1^, indicating that they were still calcites (Fig. 6i,l,o). And those dumbbell-shaped calcites were also comprised of small calcite crystals under higher magnification, just like the crystals formed with the addition of the lower concentration of rPfY2 (Fig. 6n). Those results demonstrated that rPfY2 could directly control the growth and morphologies of calcite, suggesting its significance during the biomineralization process.

**Figure 7:**
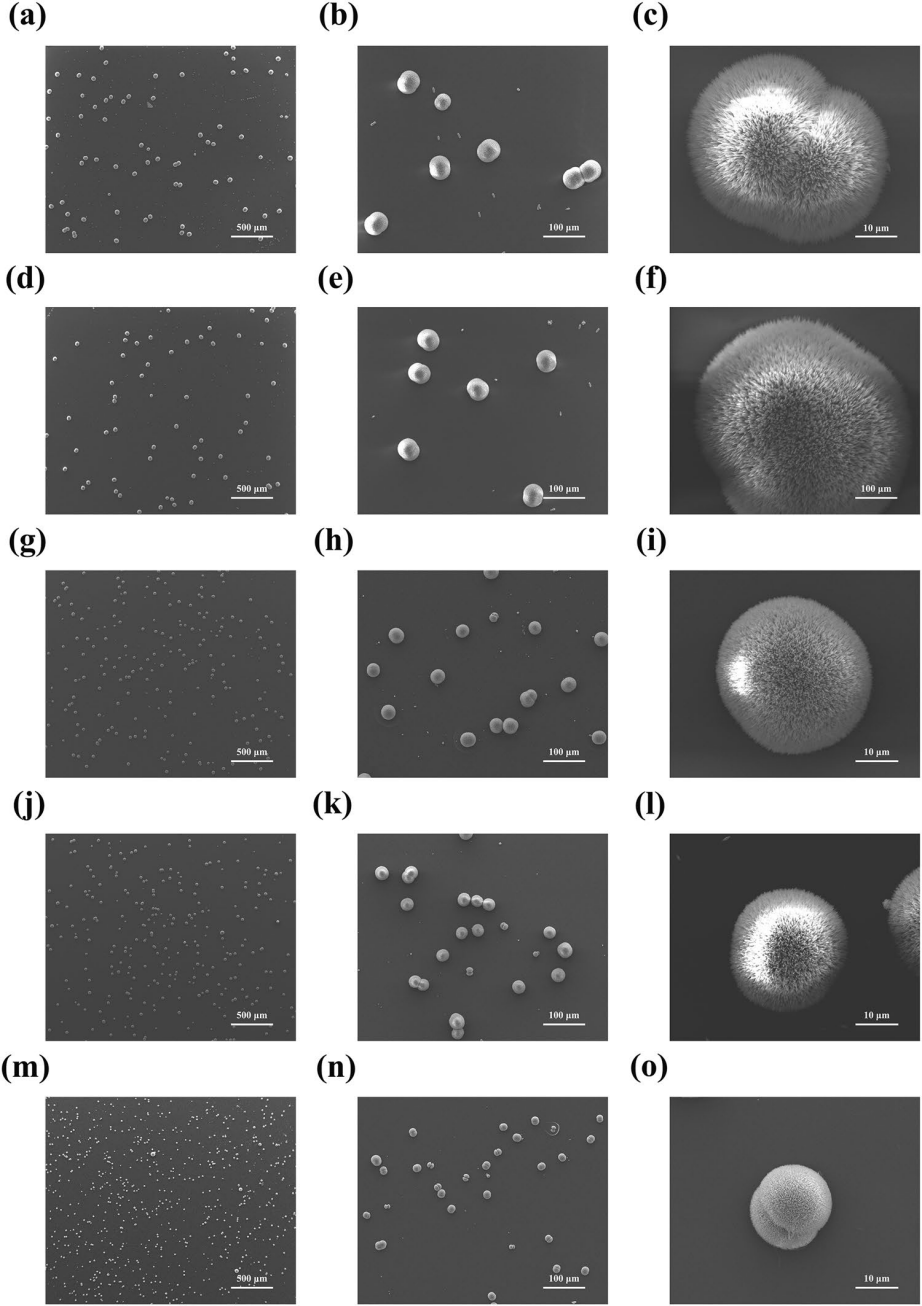
SEM images of *in vitro* aragonite crystallization in the presence of rPfY2. Crystals grown in the presence of 80 μg/ml BSA (**a**–**c**); 80 μg/ml MBP (**d**–**f**); 30 μg/ml rPfY2 (**g**–**i**); 50 μg/ml rPfY2 (**j**–**l**); 80 μg/ml rPfY2 (**m**–**o**). The middle and right columns were high-magnification images of the associated crystals.

Then magnesium was introduced into the crystallization system to induce the formation of aragonite, the main component of the nacreous layer. The standard aragonites were deposited crystals mixed with needle-like spindles, however, the addition of rPfY2 did not change the morphology or crystal form (Fig. 7). The functions of rPfY2 mainly focused on affecting the crystal number and size. The size of aragonite crystals became smaller and smaller with the increasing amount of rPfY2, from 30 μg/ml to 80 μg/ml (Fig. 7g–o). In the meantime, the number of the crystals also increased as the concentration of rPfY2 increased. As a result, we suspected that there was a strong possibility that rPfY2 could participate in the nucleation of aragonite crystallization.

Taken together, rPfY2 could both affect the crystallization process of calcite and aragonite *in vitro*, indicating its important and dual roles during biomineralization.

### *In vitro* inhibitory activity of rPfY2 on calcium carbonate precipitation

**Figure 8:**
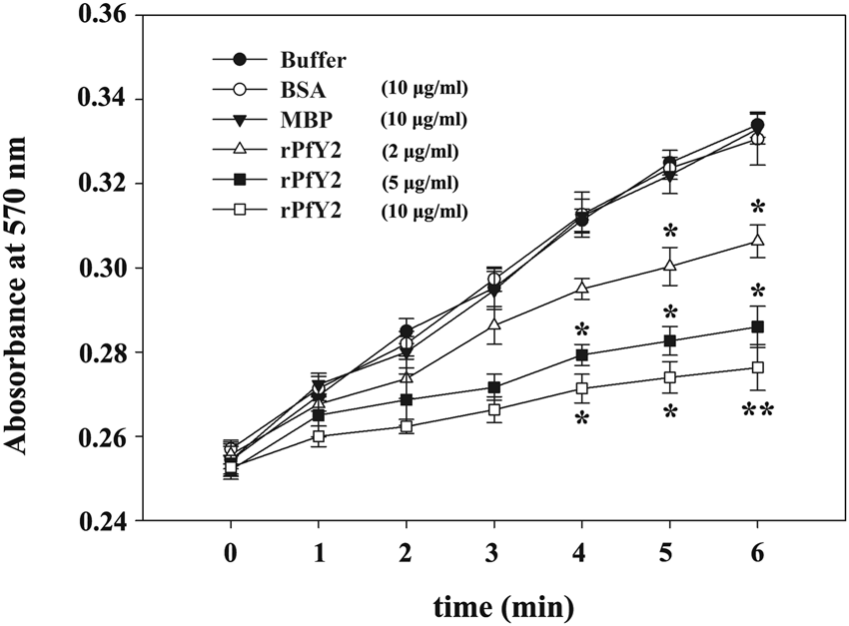
*In vitro* inhibition of rPfY2 on CaCO_3_ crystallization precipitation. CaCO_3_ precipitation rate was indicated by the absorbance of crystals at 570 nm. 2 μg/ml rPfY2 was effective (*p* < 0.05) to inhibit the precipitation rate 5 min after reaction compared with the buffer group (negative control group) and this inhibitory activity was dose-dependent. *p < 0.05; **p < 0.01.

Engagement in calcium carbonate precipitation is another important function of matrix proteins. And this crystallization rate was measured by the absorbance at 570 nm. This value in all control groups gradually increased and reached ~0.33 in 6 minutes (Fig. 8). The absorbance changed significantly after adding rPfY2, yet the changing trends of MBP group and BSA group were same as the negative control (buffer). The highest absorbance value did not exceed 0.31 with the addition of 2 μg/ml of rPfY2. In three control groups, the absorbance increased gradually as time extended, while it kept reducing at every minute as the amount of rPfY2 increasing. Moreover, there was a noticeable positive correlation between the reducing rate and the concentration of rPfY2. The precipitation rate was effectively inhibited 5 min after the addition of 2 μg/ml rPfY2 (*p* = 0.044), while 10 μg/ml rPfY2 could significantly inhibited this rate only 4 min after the reaction (*p* = 0.039). In addition, the *p* value of higher-concentration rPfY2 group was smaller at the same moment. Therefore, this inhibitory activity was dosage-dependent.

**Figure 9:**
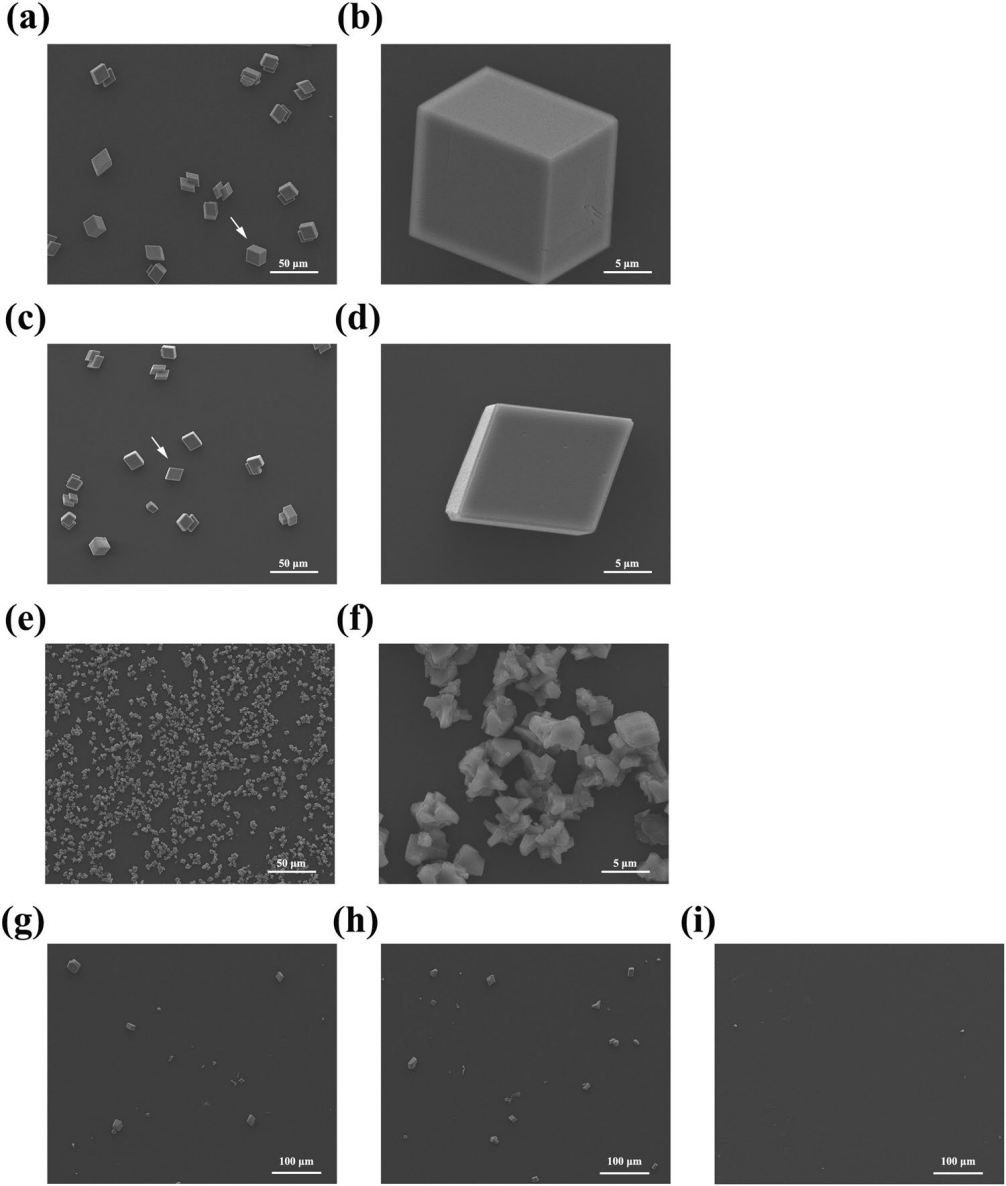
SEM images of crystals formed in the crystal growth inhibition experiments. SEM pictures of crystals grown in the presence of 10 μg/ml BSA (**a**,**b**); 10 μg/ml MBP (**c**,**d**); 10 μg/ml rPfY2 (**e**,**f**). (**b**,**d**,**f**) were the amplifications of the crystals indicated by arrows in (**a**,**c**,**e**), respectively. The final concentration of CaCl_2_ and NaHCO_3_ was 50 mM in (**a**–**f**) and 5 mM in (**g**–**i**). (**g**) 10 μg/ml BSA group; (**h**) 10 μg/ml MBP group; (**i**) 10 μg/ml rPfY2 group. Almost no crystal formed in the presence of 10 μg/ml rPfY2 when the concentration of Ca^2+^ reduced to 5 mM.

After crystallization for 24 h, we observed the crystals via SEM and found that the crystal size in the addition of 10 μg/ml rPfY2 was much smaller than those compared with the control group (Fig. 9a–f), when the final concentration of CaCl_2_ and NaHCO_3_ was 50 mM. What’s more, the edges and corners of the rhombohedral calcite were inhibited at the presence of rPfY2. The length of the crystals was 2–3 μm (Fig. 9e,f) on average while the length of calcite in control groups was 6–7 μm. Moreover, the number of the crystals increased with the addition of rPfY2, indicating that PfY2 might participate in the nucleation process of crystallization. Nevertheless, when the final concentration of CaCl_2_ and NaHCO_3_ reduced to 5 mM, almost no obvious crystals formed compared with the normal control group (Fig. 9g–i). Altogether, rPfY2 could significantly inhibit the calcium carbonate precipitation rate *in vitro* followed by an obvious concentration gradient effect. We speculated that PfY2 might be a negative regulator during calcium carbonate crystallization.

### Influences of rPfY2 on transition of ACC to stable crystals

**Figure 10:**
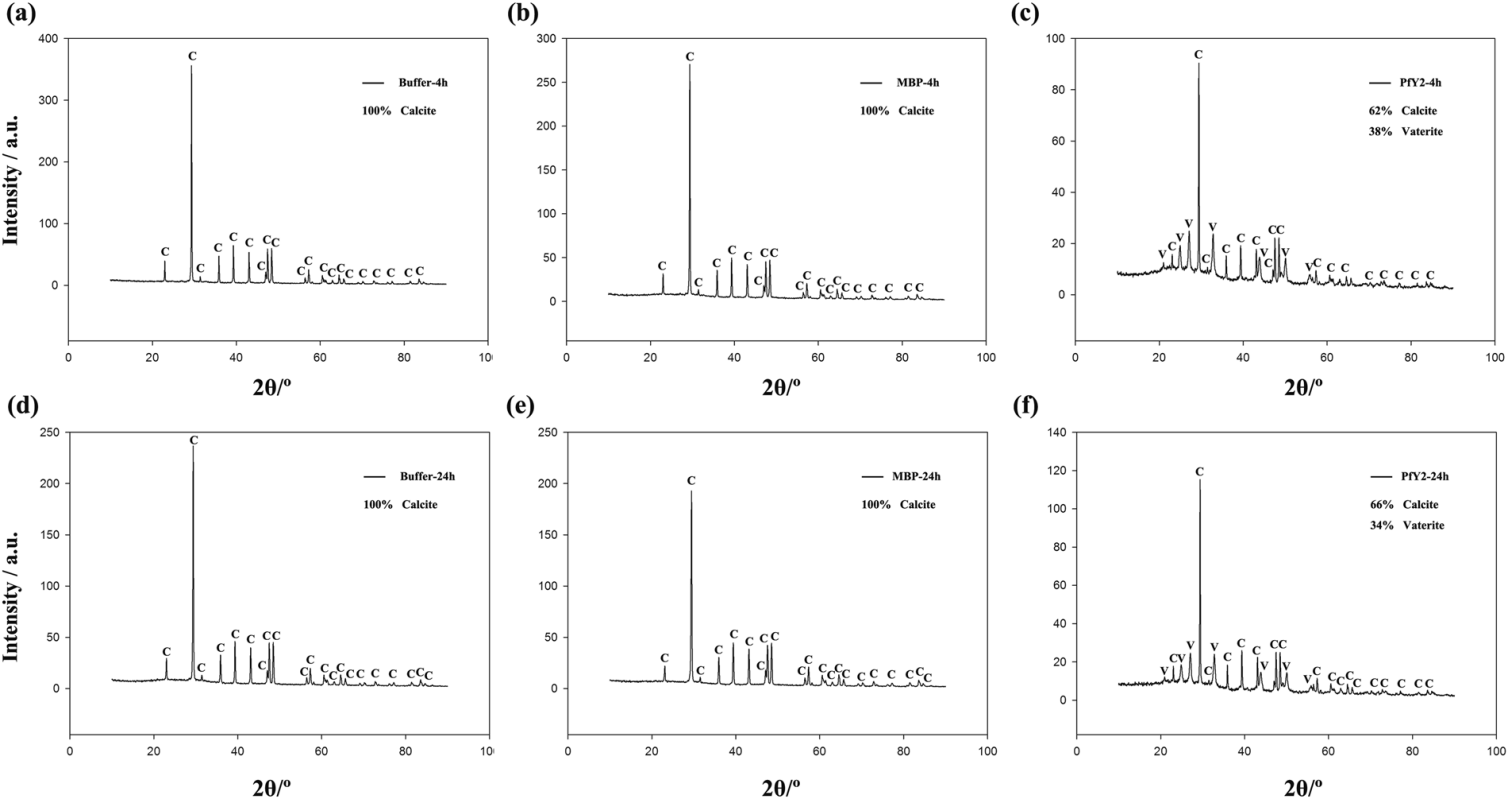
The effects of rPfY2 on ACC transition to calcite. XRD was performed to detect the percentage of the crystals ACC transformed to. The final concentration of rPfY2 was 50 μg/ml. Equivalent of MBP was added to the reaction system as control.

ACC is the precursor of predominant anhydrous polymorphs, calcite, vaterite and aragonite^3–5^. We performed the ACC transition assay to figure out the roles PfY2 played during the transition of ACC to stable crystals by X-ray diffractometer. All the ACC transformed to calcite after 4 h in the negative control group and MBP group (Fig. 10a,b), while rPfY2 disturbed this process. Only 62% of the ACC turned into calcite, 38% of them transformed to vaterite with the addition of 50 μg/ml rPfY2 after 4 h (Fig. 10c). And there was no vaterite detected in neither of the control groups. Furthermore, this ratio stayed almost unchanged after 24 h (Fig. 10f), indicating that rPfY2 could stabilize the formation of vaterite therefore inhibit the transition from ACC to calcite.

**Figure 11:**
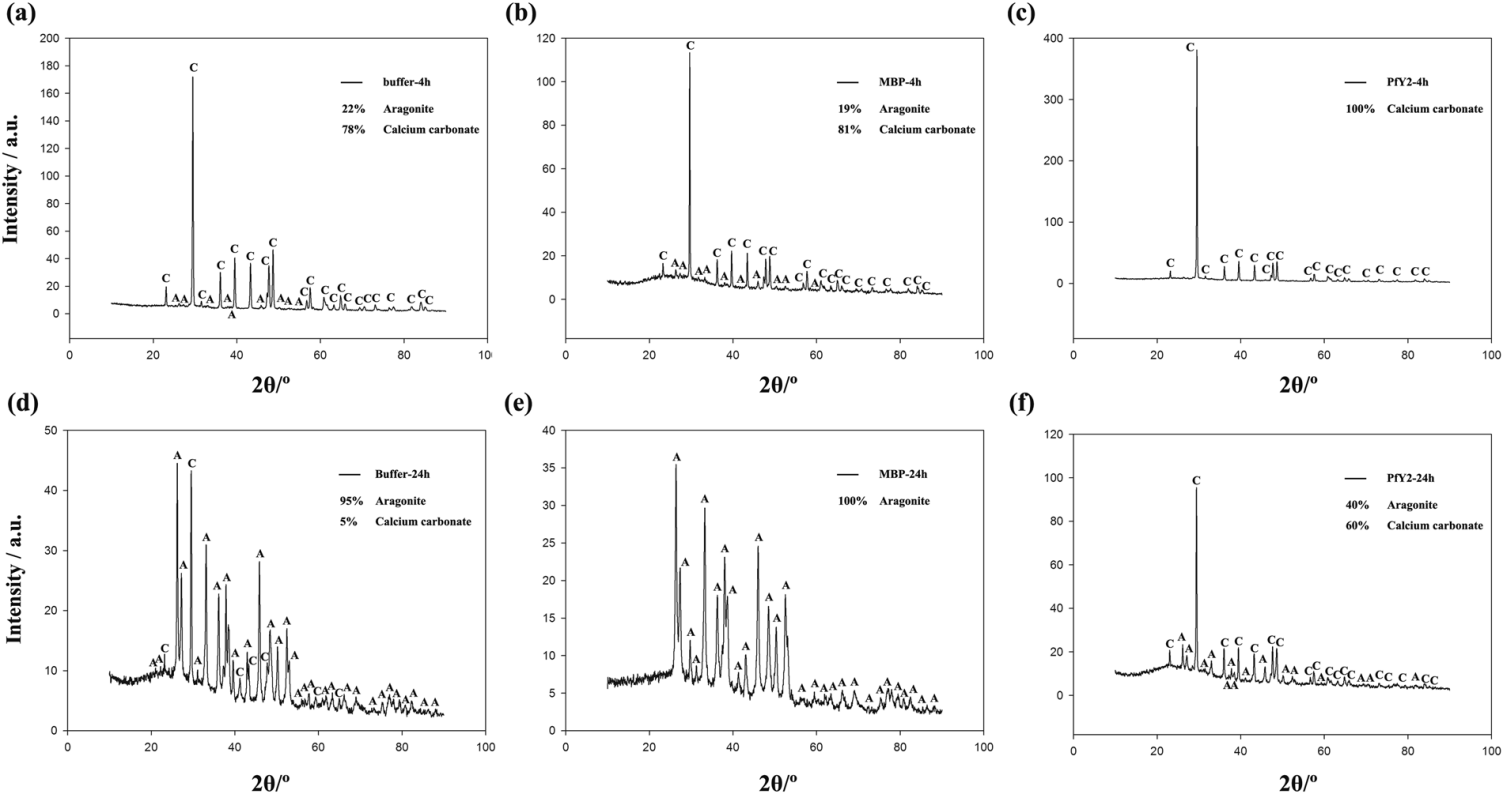
The effects of rPfY2 on ACC transition to aragonite. XRD was performed to detect the percentage of the crystals ACC transformed to. The final concentration of rPfY2 was 50 μg/ml. Equivalent of MBP was added to the reaction system as control.

With 50 mM magnesium, nearly 20% of the total ACC transformed to aragonite in both MBP and negative control group after 4 h of crystallization, and the remaining crystals were still calcium carbonate (Fig. 11a,b). When 50 μg/ml rPfY2 was added to the crystallization system, aragonite was excluded in deposited crystals and replaced by calcium carbonate (Fig. 11c). After 24 h of crystallization, nearly all of the ACC transformed to aragonite in MBP and negative control group (Fig. 11d,e). However, the introduction of PfY2 inhibited this process, and only 40% of the ACC transformed to aragonite (Fig. 11f), suggesting that rPfY2 stabilized calcium carbonate and inhibited the transition of ACC to aragonite.

## Discussion

PfY2 was identified as a potential matrix protein candidate via microarray of the global gene expression profiles during larval development in *P*. *fucata* in our previous research. Like most known genes involved in biomineralization, including nacrein, pearlin, ACCBP, KRMP etc., PfY2 showed similar expression pattern under which all of them were up-regulated dramatically in juveniles^17^. Correspondingly, the initial shell with classic structure began to form and grow after entering this stage, which is closely associated with the activation of the expression of these genes. A combination of genomic and biochemical analyses indicated that PfY2 was highly expressed in both mantle edge and mantle pallial (Fig. 1b, Suppl. Fig. S2b). According to previous studies, proteins in the EDTA-soluble fraction are known to localize within calcium carbonate crystals and responsible for regulating their morphology, nucleation, phase and orientation. While proteins in the EDTA-insoluble fraction are believed to localize around calcium carbonate crystals and participate in the formation of the shell framework^10, 18, 19, 40–43^. PfY2 was detected in EDTA-soluble fraction from both prismatic layer and nacreous layer (Fig. 1e), suggesting its dual roles during the shell formation process of *P*. *fucata* ^23^.

We analyzed the function of PfY2 in *P*. *fucata* adults *in vivo* by shell notching and RNAi. Notching brings damage to shell, mimicking the shell formation process in which biomineralization-related genes are being upregulated. The gene expression level of PfY2 increased at 12 h after notching, reaching the peak at 36 h and then with a slight decrease in the following hours. Generally, the expression pattern of PfY2 implied its participation and regulation on CaCO_3_ precipitation. As shown by the rate determination experiment of CaCO_3_ precipitation *in vitro* (Fig. 8) and the transition of ACC to stable crystals (Figs 10 and 11), PfY2 clearly affected crystal deposition and inhibited CaCO_3_ crystallization. Based on these performances, PfY2 presumably functioned as a negative regulator during biomineralization. Since shells cannot grow infinitely, there must be some “safety guard” that slows down this process, functioning as an inhibitor to limit crystal growth and regulate their morphologies or orientation. Our lab found extrapallial fluid proteins and some other matrix proteins, like PfN44, played paradoxical roles in both CaCO_3_ crystallization and shell biomineralization balance^9, 38^. As a result, we speculated that the small increase of the “negative regulator” PfY2 during shell notching mainly contributed to the balance of shell growth. RNAi assay showed that after the interference of PfY2, the “negative regulator”, many small nacreous blocks with the overgrowth of crystals emerged on the surface of nacreous layer (Fig. 4e–h). Since shell formation is under the control of many different matrix proteins; there is no doubt that they work together to maintain the balance of shell growth and reparation. Therefore, the porous structures in prismatic layer might be the results of shell overgrowth or shell defects made by the mess of the matrix protein regulatory network.

According to ACC transition experiment, the percentage of vaterite in transition system was almost constant either 4 h or 24 h after the introduction of rPfY2 (Fig. 10c,f), coinciding with rPfY2′s inhibition effects on CaCO_3_ precipitation rate. Nevertheless, vaterite has not been found in our animal model *P*.*fucata* while some exceptions have been reported in other close-related species. A new morphology of vaterite was confirmed in lackluster pearls of *Hyriopsis cumingii* ^44^. Moreover, vaterites also formed in the abnormal shells of *Corbicula fluminea* ^45^. In these few reported cases, vaterite formation in mollusks is usually closely related to the unusual or abnormal biomineralization. Thus, we infer that vaterite is a kind of transient phase during shell formation and only when its further transformation is inhibited could it be detected. This might be the reason why we couldn’t find vaterite in normal shell of *P*. *fucata*. And we could infer that maybe PfY2′s regulation on crystallization relied on its stabilization property on this intermediate vaterite phase.

Studies conducted on CaCO_3_ mineral interaction domains have identified several key criteria which define a CaCO_3_ mineral recognition sequence including the presence of hydrogen-bonding donor/acceptor residues (i.e., Ser, Tyr, Thr, Asn, Gln, Lys, Arg) representing putative sites for carbonate and/or mineral surface water interactions^46–49^. rPfY2 is an Asn-rich protein, based on which we supposed that rPfY2 bound with CO_3_^2−^ at the first place to locally recruit ions for nucleation followed by binding on the surfaces of CaCO_3_ micro-particles thus inhibiting the growth and aggregation of particle. That may be the reason why A_570_ value in the rPfY2 group was lower than that compared with the control group (Fig. 8). This could also explain the micro-particles emerged in calcite crystallization experiment in Fig. 6g–n. Under this hypothesis, when existed in a super-saturation system, rPfY2 might increase local concentration of ions to promote nucleation process. By the time CaCO_3_ nucleus formed and grew into a certain diameter, then rPfY2 bound to the surfaces of CaCO_3_ particles, forming steric hindrance to slow down the ions absorption process and inhibit the aggregation of CaCO_3_ particles, thus the crystal growth process was inhibited (Figs 7 and 9a–f). In addition, we need to assure that when the concentration of Ca^2+^ was low enough (5 mM), rPfY2 could inhibit the growth of almost all crystals (Fig. 9g–i). While increasing the concentration of rPfY2 in ACC transforming experiments, the binding and inhibitory abilities became stronger, which might restrict the transforming from vaterite to calcite so that the unstable vaterite phase could exist for a long time in Fig. 10.

These results together confirmed that the “safety guard” PfY2 participating in CaCO_3_ crystallization functioned as a negative regulator during this process and shell formation. This could also explain that the expression of PfY2 was upregulated in juveniles rather than D-shaped stage due to its inhibition on ACC transition, which might lead to the disruption of the formation of prodissoconch I. Therefore, PfY2 plays a critical role in the sophisticated regulation of shell formation and biomineralization.

## Conclusion

The novel matrix protein PfY2, located in EDTA-soluble fraction from both prismatic layer and nacreous layer, plays important roles during shell biomineralization. Our results in this work show that PfY2 is a crucial matrix protein participating in the biomineralization regulation. And this is significant to reveal the inhibition mechanism during shell formation and may bring us new inspiration on the synthesis of artificial nacre.

## Electronic supplementary material

Suppplementary Information
