# Supplementary material for: A Novel Matrix Protein, PfY2, Functions as a Crucial Macromolecule during Shell Formation
Source: Sci Rep. 2017 Jul 20;7:6021. doi: 10.1038/s41598-017-06375-w (PMC5519542; doi:10.1038/s41598-017-06375-w)
Supplement: Supplementary file 1 — Suppplementary Information [file 41598_2017_6375_MOESM1_ESM.pdf]

Supplementary information

A Novel Matrix Protein, PfY2, Functions as a Crucial Macromolecule during Shell Formation

Yi Yan[1], *Dong Yang*[1], *Xue Yang*[1],

Chuang Liu[1], *Jun Xie*[1], *Guilan Zheng*[1], *Liping Xie*[1, 2, *], *Rongqing Zhang*[1, 2, 3, *]

[1] Institute of Marine Biotechnology, School of Life Sciences, Tsinghua University, Beijing 100084 China,

E-mail: lpxie@mail.tsinghua.edu.cn (LX); rqzhanglab@mail.tsinghua.edu.cn (RZ)

[2] Protein Science Laboratory of the Ministry of Education, Tsinghua University, Beijing 100084 China

[3] Department of Biotechnology and Biomedicine, Yangtze Delta Region Institute of Tsinghua University, Jiaxing 314000 China

The expression pattern of PfY2 during the development from fertilized oocyte to juveniles.

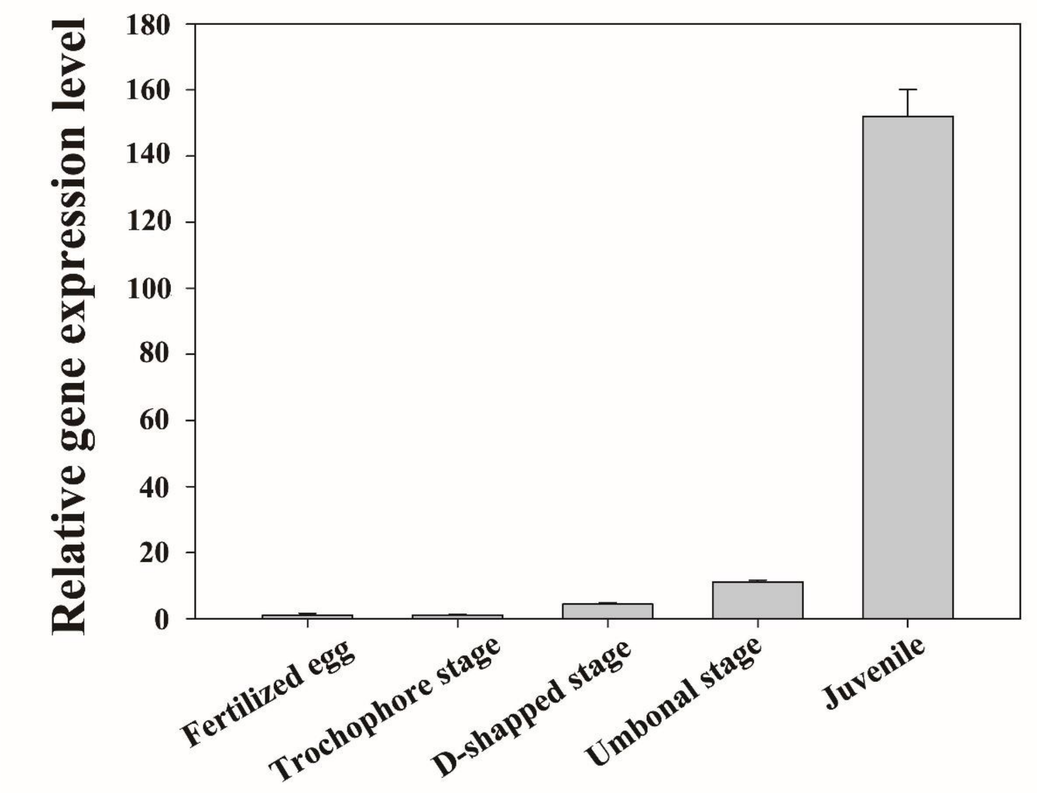

Figure S1. Gene expression level of PfY2 during the six developmental stages. Values for RT-PCR are means ± standard deviation of three replicates. Expression of fertilized egg gave a relative value of 1.0.

PfY2's Specific Expression Site in Mantle Tissue in *P. fucata.*

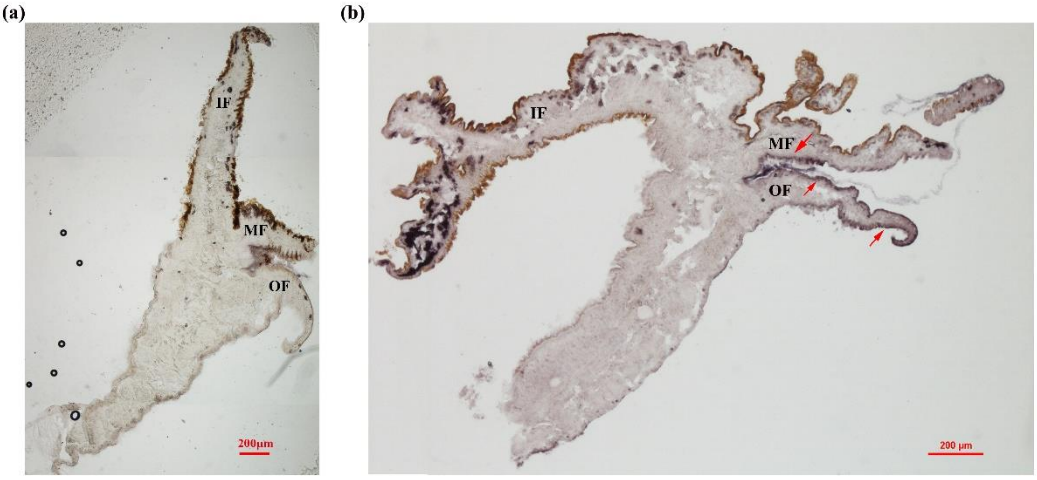

Figure S2. *In situ* hybridization of PfY2 in mantle tissue. Negative control group showed no significant signals (a). Positive signals were indicated by arrow (b). OF, outer fold; MF, middle fold; IF, inner fold. Scale bar, 200 μm.

The Identification of Recombinant protein rPfY2 with MBP Tag

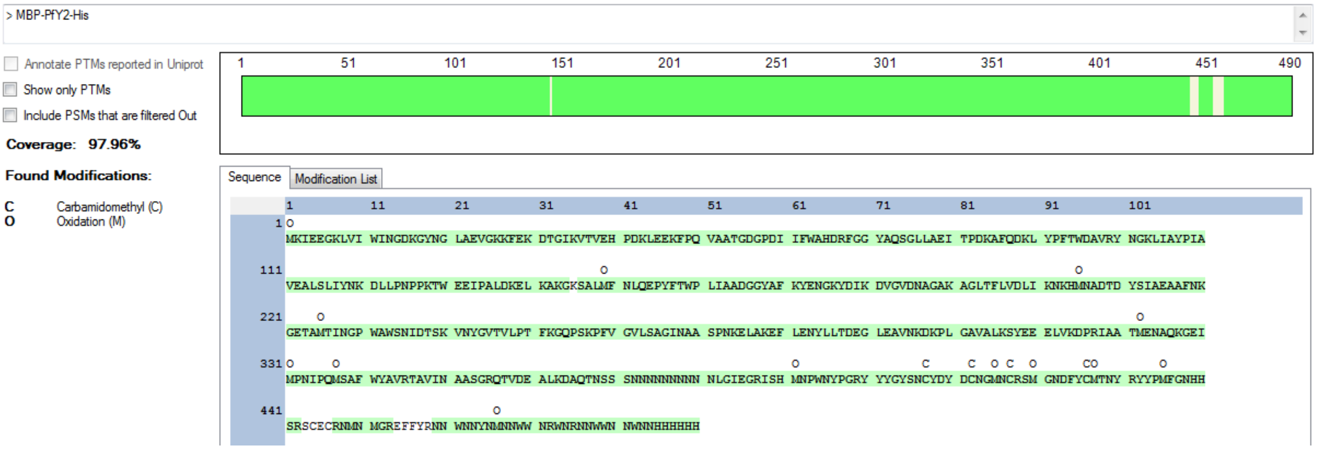

Figure S3. Identification of recombinant rPfY2 protein sequence. The sequence of rPfY2-MBP with a His_6-tag on C-terminal was confirmed by mass spectrum.

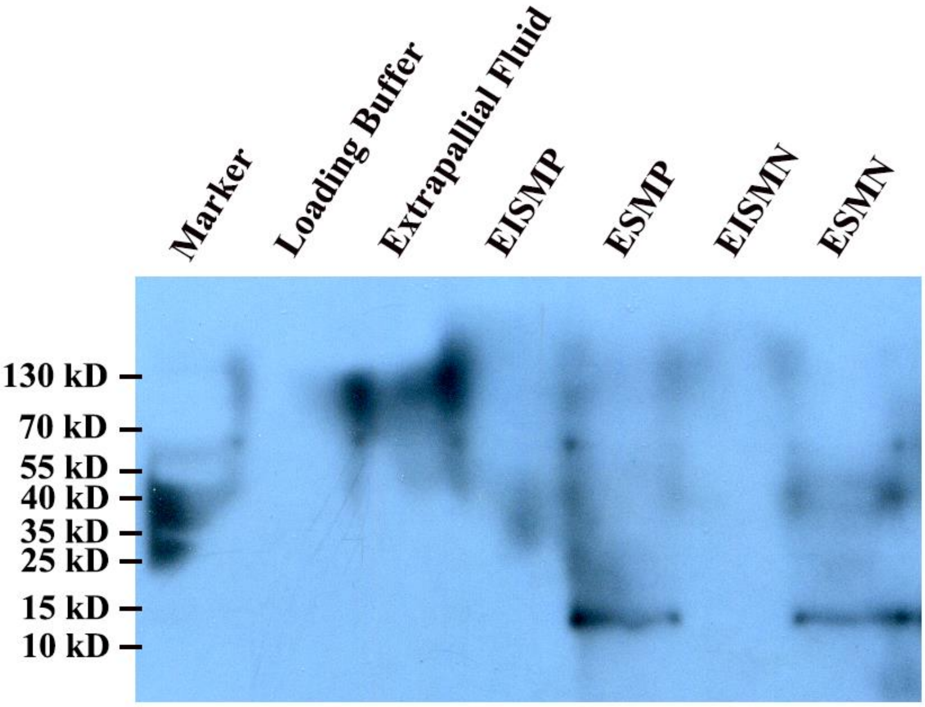

Figure S4. The primary gel of the identification of PfY2 as a matrix protein. The marker was underexposed. EISMP, EDTA-insoluble fraction from prismatic layer; ESMP, EDTA-soluble fraction from prismatic layer; EISMN, EDTA-insoluble fraction from nacreous layer; ESMN, EDTA-soluble fraction from nacreous layer.

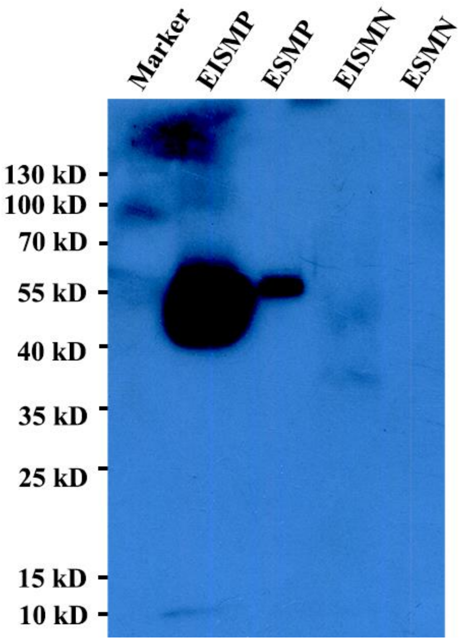

Figure S5. The primary gel of the identification of PfY2 as a matrix protein by Western Blotting in the absence of the anti-PfY2. The marker was underexposed. EISMP, EDTA-insoluble fraction from prismatic layer; ESMP, EDTA-soluble fraction from prismatic layer; EISMN, EDTA-insoluble fraction from nacreous layer; ESMN, EDTA-soluble fraction from nacreous layer.

The Expression Pattern of other Matrix Proteins after Knocking Down the Expression Level of PfY2

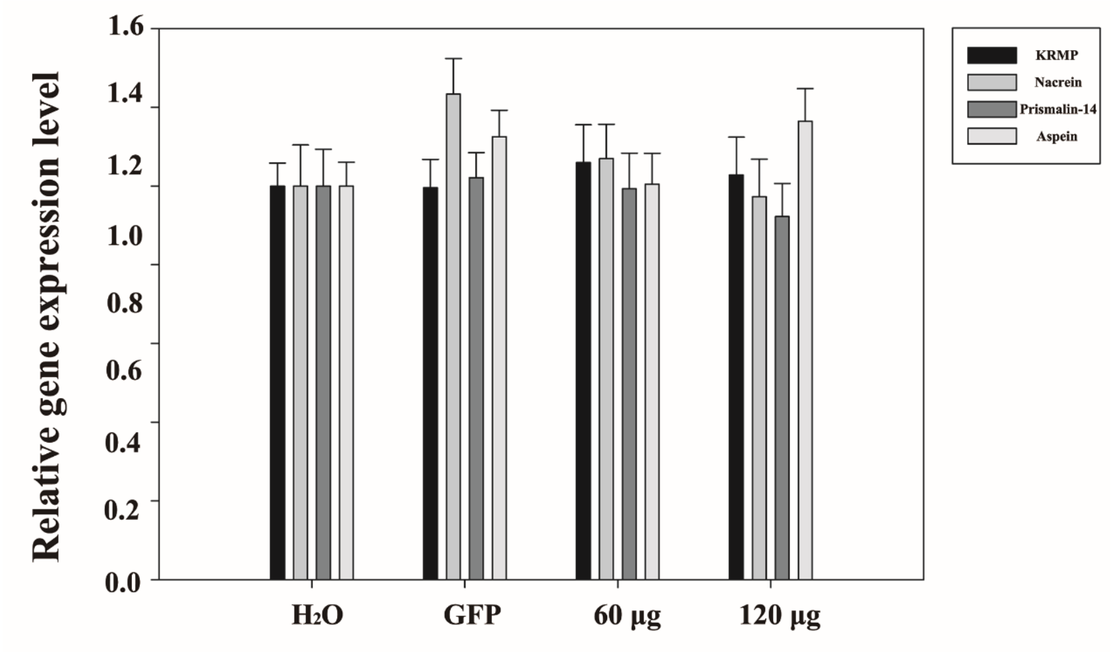

Figure S6. Gene expression levels of other matrix proteins when PfY2 was knocked down by RNAi. The expression levels were analyzed 6 days after the injection of PfY2 dsRNA and five individuals were tested from each group. In the control, the expression levels of the water-injected group were normalized to a relative value of 1.0.

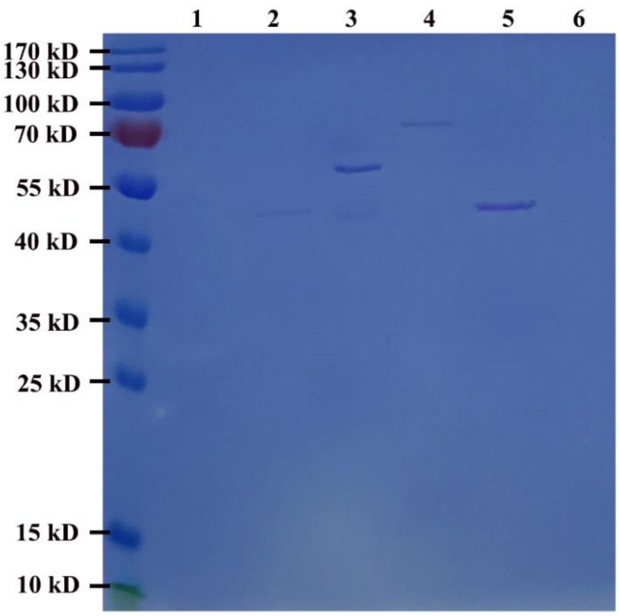

Figure S7. The primary gel of PfY2's binding properties with calcite. Lane 1-3, BSA, MBP, rPfY2 binding with calcite after flushing with water and protein elution buffer three times each, respectively. Lane 4-6, residual unbound BSA, MBP, rPfY2 in 1st wash fraction after incubation with calcite, respectively.

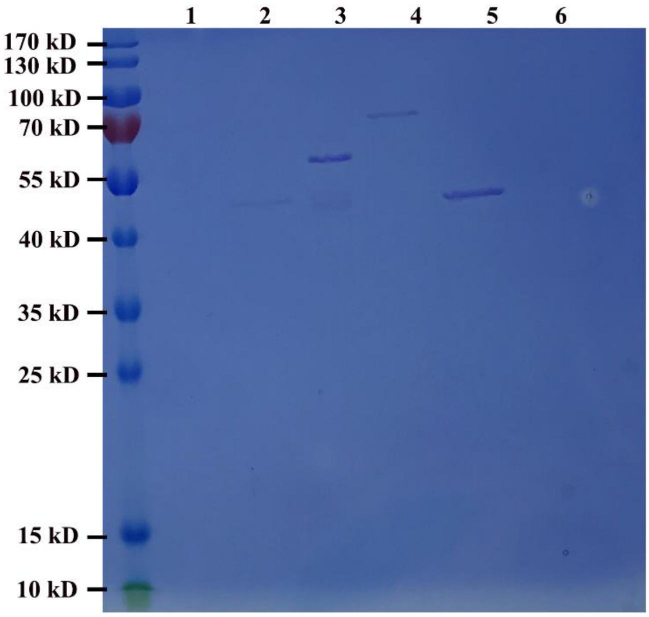

Figure S8. The primary gel of PfY2's binding properties with aragonite. Lane 1-3, BSA, MBP, rPfY2 binding with aragonite after flushing with water and protein elution buffer three times each, respectively. Lane 4-6, residual unbound BSA, MBP, rPfY2 in 1st wash fraction after incubation with aragonite, respectively.
